# Customized Efficient Neural Network for COVID-19 Infected Region Identification in CT Images

**DOI:** 10.3390/jimaging7080131

**Published:** 2021-08-04

**Authors:** Alessandro Stefano, Albert Comelli

**Affiliations:** 1 Institute of Molecular Bioimaging and Physiology, National Research Council (IBFM-CNR), 90015 Cefalù, Italy; alessandro.stefano@ibfm.cnr.it; 2 Ri.MED Foundation, 90133 Palermo, Italy

**Keywords:** COVID-19, deep learning, segmentation, computed tomography, customized ENET

## Abstract

Background: In the field of biomedical imaging, radiomics is a promising approach that aims to provide quantitative features from images. It is highly dependent on accurate identification and delineation of the volume of interest to avoid mistakes in the implementation of the texture-based prediction model. In this context, we present a customized deep learning approach aimed at addressing the real-time, and fully automated identification and segmentation of COVID-19 infected regions in computed tomography images. Methods: In a previous study, we adopted ENET, originally used for image segmentation tasks in self-driving cars, for whole parenchyma segmentation in patients with idiopathic pulmonary fibrosis which has several similarities to COVID-19 disease. To automatically identify and segment COVID-19 infected areas, a customized ENET, namely C-ENET, was implemented and its performance compared to the original ENET and some state-of-the-art deep learning architectures. Results: The experimental results demonstrate the effectiveness of our approach. Considering the performance obtained in terms of similarity of the result of the segmentation to the gold standard (dice similarity coefficient ~75%), our proposed methodology can be used for the identification and delineation of COVID-19 infected areas without any supervision of a radiologist, in order to obtain a volume of interest independent from the user. Conclusions: We demonstrated that the proposed customized deep learning model can be applied to rapidly identify, and segment COVID-19 infected regions to subsequently extract useful information for assessing disease severity through radiomics analyses.

## 1. Introduction

In the era of the 2019 coronavirus (COVID-19) pandemic, medical imaging, e.g., computed tomography (CT), plays a preponderant role in the fight against this disease [1]. Specifically, there is a growing interest in the analysis of novel image segmentation techniques to improve radiomics studies related to COVID-19 [2]. Unlike qualitative approaches, in which biomedical images are inspected and interpreted visually [3], radiomics analyses a large number of parameters to identify any statistical correlation with measurable aspects of the disease to identify the most relevant features. On the other hand, radiomics is strongly linked to the identification of the target region. When the delineation is performed manually, hundreds of CT slices have to be segmented, making this an extremely time-consuming task. Moreover, and, above all, the segmentations become highly operator-dependent [4,5,6]. Radiomics obtains repeatable results only if the segmentation task is independent of the operator [7]. For this reason, automatic target identification (e.g., COVID-19 infected regions) becomes crucial to avoid bias in the feature extraction task and to ensure repeatable radiomics results [8,9]. Automated segmentation is a general key issue in medical image analysis and remains a popular and challenging research area. Although CT provides high-quality images for COVID-19 detection, the lungs can be affected by extensive patchy ground-glass regions and consolidations and may even show pleural fluid [10,11]. As a result, automated lung segmentation is a very challenging process. Furthermore, supervised segmentation algorithms, such as those proposed in our previous lung study [12], require user control and time-consuming manual corrections. Artificial intelligence (AI) can be used to overcome these limitations by achieving accurate and operator-independent identification and delineation of COVID-19 infections, facilitating subsequent image quantification for disease diagnosis and prognosis [13]. Consequently, there is a quantity of research related to enhanced AI applications on chest X-rays or CT images showing effectiveness in both follow-up assessment and COVID-19 evolution evaluation [14]. These studies indicate great potential for clinical support, but they are often overfitting, being trained using proprietary data or from a single site, i.e., [15,16]. In [15], the authors proposed a new weakly supervised learning method (Inf-Net) in which they aggregated high-level features using a parallel partial decoder for segmentation of infected regions. To improve the representation, explicit edge and implicit reverse attention were used, resulting in a dice similarity coefficient (DSC) ~ 58% on nine real CT volumes. Zhou et al. [16] integrated the spatial and channel attention mechanism in U-Net to obtain a better representation of the features. Additionally, Tversky focal loss was introduced to address the segmentation of small lesions. The two datasets used in the experiments come from the Italian Society of Medical and Interventional Radiology obtaining a DSC~83%. To overcome the issue of proprietary data or single-site data, Roth et al. [17] organized an international challenge and competition for the development and comparison of AI algorithms using public data. Consequently, this study aims to propose a customized Efficient Neural Network (ENET) to segment Covid-19 infections using the dataset provided by [17] for the experiments. Specifically, ENET was originally proposed for real-time image delineation on low-power mobile devices [18]. Subsequently, it has been used successfully for lung, aorta, and prostate segmentation tasks [19,20,21]. In the first study, a small dataset of images from CT studies of patients with idiopathic pulmonary fibrosis (IPF) was considered. IPF is a form of interstitial lung disease characterized by a continuous and irreversible process of fibrosis that causes the progressive decay of lung function leading to death. In patients with IPF, CT images are characterized by honeycombing, extensive patchy ground-glass regions with or without consolidations, presence of pleural fluid, and, of course, fibrotic regions. Quantification of the affected IPF regions is crucial for patient assessment and for the detection of parenchyma abnormalities [12]. COVID-19 has several common features with IPF; some of the features mentioned above can be identified in COVID-19 infected lungs [22]. The main difference between the two diseases is the presence of peripheral ground-glass opacities and less or no fibrosis in COVID-19 infected lungs [23]. Consequently, this study aims to investigate how a DL network used for the parenchyma segmentation task in patients with IPF can be applied, with appropriate adjustments, to detect and segment COVID-19 infected lung regions. We explore the effectiveness of the customized ENET model, namely C-ENET, compared to other state-of-the-art models, namely UNET [24] and ERFNET [25], using the online COVID-19 Lung CT Lesion Segmentation Challenge—2020 (COVID-19-20) dataset [17,26]. The results show the great potential of the proposed network in automatically identifying and segmenting COVID-19 infected subregions for the subsequent extraction of quantitative features. In this way, the methodology performed in radiomics studies could benefit from an operator-independent segmentation method capable of automatically extracting the target from COVID-19 infected lungs.

## 2. Materials and Methods

### 2.1. Dataset

**Figure 1:**
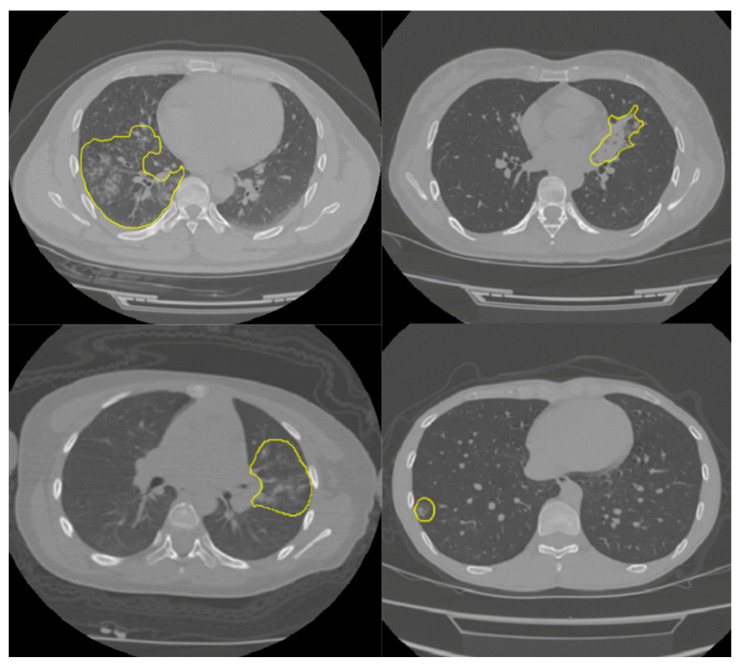
CT images showing the parenchyma with visible subregions (yellow) infected with COVID-19 disease.

In this study, the online COVID-19 Lung CT Lesion Segmentation Challenge—2020 (COVID-19-20) dataset was used to train and test our model. The dataset contains 199 lung studies with a matrix resolution of 512 × 512 and with labels provided by the Challenge [26] (four CT examples are shown in Figure 1). Dataset annotation was made possible through the joint work of the Children’s National Hospital, NVIDIA and the National Institutes of Health for the COVID-19-20 Lung CT Lesion Segmentation Grand Challenge. The aim was to identify and segment the lung subregions infected with the disease. Since DL networks require inputs of the same size for training, CT voxels were resampled to the isotropic size of 1 × 1 × 1 mm^3^ using linear interpolation. We implemented the network and the Tversky loss function [27] (see Section 2.3) using Keras with Tensorflow backend in the open-source language Python (www.python.org, accessed on 1 January 2021). We used the whole dataset in a k-fold (k = 5) cross-validation fashion, as explained in Section 2.4.

### 2.2. C-ENET

ENET is an optimized neural network implemented for the high accuracy and fast inference typically needed in the self-driving car industry. Its architecture is widely described in [18]. Briefly, it is based on building blocks of residual networks, with each block consisting of three convolutional layers. ENET is characterized by asymmetric and separable convolutions with sequences of 5 × 1 and 1 × 5. The 5 × 5 convolution has 25 parameters while the corresponding asymmetric convolution has 10 parameters to reduce the size of the network. This network has been applied for parenchyma segmentation in patients with IPF that presents several similarities to COVID-19 disease [19]. Now, we customize the proposed network to apply it in COVID-19 infected lungs. The difference between the two network architectures (ENET and customized-ENET, namely C-ENET) is shown in Table 1. In stage 3, the output of 128 × 64 × 64 was replaced by an output of 256 × 64 × 64. This implies a DSC distribution on average greater and with less variability than ENET, as shown in Section 3.

### 2.3. Loss Function

In CT images, the number of voxels belonging to the target is small compared to the number of voxels belonging to the background. The COVID-19 lung regions infected are very small compared to the background which can be composed of many different organs or tissues characterized by a wide range of Hounsfield unit (HU) values. DL suffers from this problem as it has difficulty learning a reliable feature representation of the target class. Consequently, it tends to identify most CT regions as belonging to the background class. To overcome this issue, as reported in [28], the Tversky loss function [27] is used for the weight adjustment of false positives (FPs) and false negatives (FNs), unlike the DSC that is the most used DL loss function. Specifically, DSC is the harmonic mean of FPs and FNs and weighs both equally. Vice versa, the Tversky loss is defined as follows:

(1) $$T\left(\alpha \;\beta \right)=\frac{\sum_{i=1}^{N}p_{0i}\;g_{0i}}{\sum_{i=1}^{N}p_{0i}\;g_{0i}+\alpha \sum_{i=1}^{N}p_{0i}\;g_{1i}+\beta \sum_{i=1}^{N}p_{1i}\;g_{0i}}$$

where *p*_0*i*_ is the probability of voxel I being part of the target, and *p*_1*i*_ is the probability of it belonging to the background. The ground truth training label *g*_0*i*_ is 1 for target and 0 for everything else, and vice versa for the label *g*_1*i*_. By adjusting the parameters, 𝛼 and 𝛽, the trade-off can be controlled between FPs and FNs. To obtain DSC, set 𝛼 = 𝛽 = 0.5. Setting β’s > 0.5 weight recall higher than precision by placing more emphasis on FNs in the slices with small target regions.

### 2.4. Training

In AI studies, images are divided into three datasets: (i) training, (ii) validation, and (iii) testing. However, if a small number of images are available for training, as is often the case in the biomedical field, the k-fold cross-validation strategy can be used. In this way, CT studies are divided into k folds: one fold is used as the validation set and the remaining folds are used as the training set. This process is repeated k times using each fold as the validation set and the other ones as the training set. As a result, we used a 5-fold by randomly dividing the CT studies into 5 folds of 40 or 39 studies. In this way, 5 models were trained differently. The performance result was obtained by averaging the performance results of the validation datasets of the 5 models.

### 2.5. Experimental Details 

We implemented DL networks using the open-source programming language Python and a high-end HPC system equipped with GPU (NVIDIA QUADRO P4000 with 8 GB of RAM, 1792 CUDA Cores). We used a learning rate of 0.0001 for ENET and C-ENET, and 0.00001 for ERFNET and UNET with the Adam optimizer, while a batch size of 8 slices was set for all experiments [19,20,21]. During the training task, a maximum of 100 epochs was allowed. The criterion of stopping of the Tversky loss function was characterized by α = 0.3, and β = 0.7. If the training loss did not decrease for 10 consecutive epochs, the training was stopped. We also applied the data augmentation technique by randomly rotating (20), translating in both x = 0.2 and y = 0.3 directions, and applying shearing (20), horizontal flip, zooming (0.4) and elastic transform (α = 0.8, and σ = 0.2). In addition, standardization and normalization of data were applied to converge faster and avoid numerical instability and too large weights.

### 2.6. Evaluation Metrics

The performance result of the proposed approach was obtained using DSC, sensitivity, volume overlap error (VOE), volumetric difference (VD), and positive predictive value (PPV) calculated as mean, standard variation (std), and confidence interval (CI) [29]. The DSC measures the spatial overlap between the reference volume and the segmented volume. It ranges between 0% (no overlap) and 100% (perfect overlap) and is calculated as:

DSC= (2 × 𝑇𝑃)/(2 × 𝑇𝑃 + 𝐹𝑃 + 𝐹𝑁) × 100%(2)

where *TP*, *FP*, and *FN* are the number of true positives, false positives, and false negatives respectively. 

Sensitivity is the true positive rate and is defined as:

Sensitivity = (𝑇𝑃)/(*TN* + 𝐹𝑁) × 100% (3)

where *TN* is the number of true negatives.

Finally, VOE, VD, and PPV are defined respectively as:

VOE = 1 − (*TP*)/(*TP* + *FP* + *FN*) × 100% (4)

VD = (*FN* − *FP*)/(2*TP* + *FP* + *FN*) × 100% (5)

PPV = (𝑇𝑃)/(*TP* + 𝐹𝑁) × 100% (6)

To test the significance of differences between the DL algorithms’ performance, an analysis of variance (one-way ANOVA) with Tukey HSD (honestly significant difference) was used as multiple comparison correction techniques on the DSC. A *p*-value ≤ 0.05 was considered statistically significant.

## 3. Results

We applied the proposed segmentation approach to identify and contour COVID-19 infected regions and we provided a comparison with the results of the original ENET, UNET, and ERFNET. Table 2 shows the performance evaluation using the k-fold strategy. C-ENET showed the highest DSC (74.83 ± 11.18%), while ENET and ERFNET achieved a DSC of 72.28 ± 13.44% and 54.23 ± 18.64%, respectively. 

**Figure 2:**
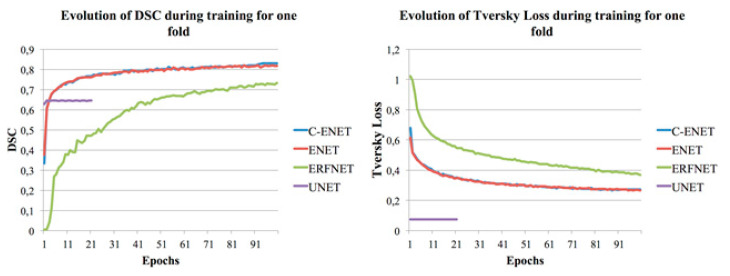
Training DSC and Tversky loss plots for C-ENET, ENET, ERFNET and UNET.

UNET [24] was unable to achieve any convergence after more than twenty-one epochs (with reference to Figure 2 where the training DSC and Tversky loss function [27] plots are shown for one-fold and for each considered algorithm), unlike our previous studies whose purpose was the segmentation of the whole lung, aorta, and prostate [19,20,21]. Specifically, during the training, the network immediately gave a low value of Tversky loss (0.075) and a value of DSC = 62.79%. The network maintained the same values after twenty-one epochs, consequently, the training was stopped (the training loss did not decrease for 10 consecutive epochs). Although the network maintained low Tversky loss values, it was unable to learn; in fact, by testing the validation dataset, we obtained a DSC of 0%. A further test was performed with a modified version of UNET where all 3 × 3 convolutions were replaced by larger 5 × 5 convolution operators as reported in [19], and similar results were obtained. For this reason, UNET was not included in Table 2. Figure 2 also shows how C-ENET and ENET models outperformed the other two models. Specifically, both models achieved a training DSC greater than 80% in less than 50 epochs.

At the analysis of variance, the *p*-value corresponding to the F-statistic of one-way ANOVA was less than 0.05, suggesting that one or more methods were significantly different (see Table 3). 

**Figure 3:**
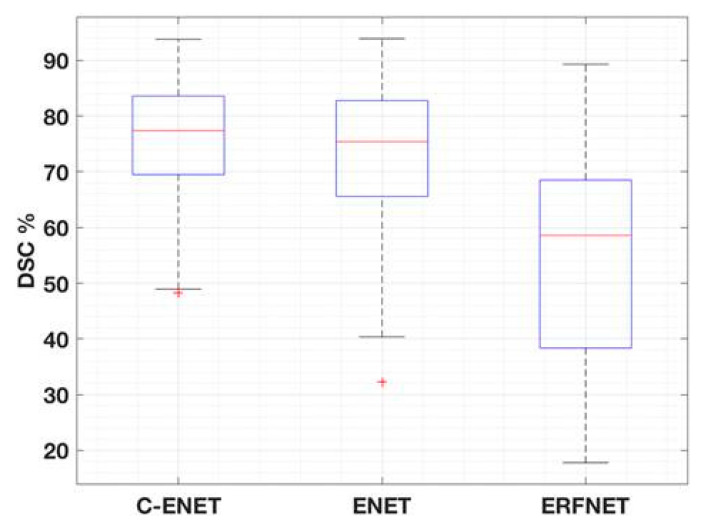
DSC-based boxplots of the different DL networks.

**Figure 4:**
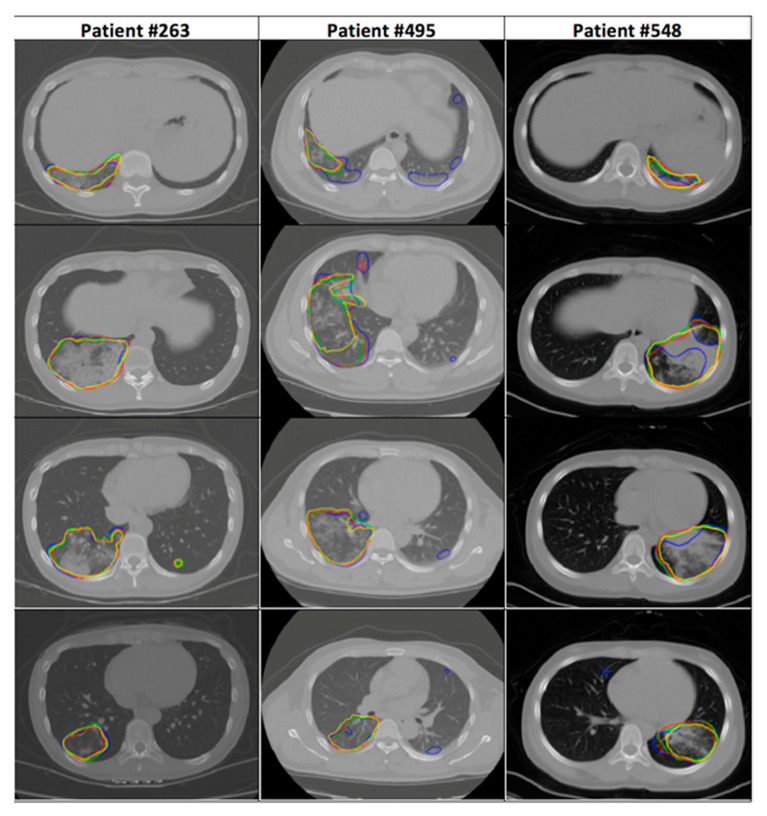
CT images showing parenchyma with visible subregions (yellow for the gold standard, red for C-ENET, green for ENET, and blue for ERFNET,) infected with COVID-19 disease.

Table 4 shows the multiple comparison using the Tukey HSD correction technique. C-ENET was significantly different from ERFNET but not from ENET. However, DSC-based boxplots were also calculated (with reference to Figure 3) to further evaluate the differences in algorithm performance. Although C-ENET and ENET methods obtained very good performance, C-ENET showed a DSC distribution on average greater and with less variability than ENET. Consequently, it can be assumed that C-ENET focuses on higher performance values. Examples of obtained results are shown in Figure 4.

Finally, Table 5 shows the computational complexity of the three DL networks. In particular, we estimated the time taken to complete a CT study by considering the average time required to obtain the output for each study. Using the GPU (NVIDIA QUADRO P4000, 8 GB VRAM, 1792 CUDA Cores) about 2–4 s were required for each network. When calculations were performed on a CPU (Intel(R) Xeon(R) W-2125 CPU 4.00 GHz processor), DL networks required less than 17 s.

## 4. Discussion and Conclusions

In this study, we investigate user-independent strategies to support clinicians with the automatic identification and delineation of COVID-19 infected regions in CT images. Our main goal is to devise an algorithm capable of satisfying the growing need for an efficient, repeatable, and real-time segmentation approach without any user supervision, unlike our previous studies [12,30,31]. Identifying the volume of interest is the first task of a radiomics analysis. Accurate delineation is essential to avoid mistakes in the calculation of features. Furthermore, when target identification is performed manually, the results are characterized by high variability [7]. For this reason, with the advent of COVID-19 pandemic, there is a growing interest in the use of automated and operator-independent image segmentation strategies to detect SARS-CoV-2 infection in the parenchyma. The DL methods are more efficient than the classical statistical approaches, obtaining better performance in the classification of images and in the segmentation of several anatomical districts. In this study, we explore the performance of ENET, as proposed in [19] for IPF patient studies, and appropriately modified for our purpose, henceforth referred to as the C-ENET (with reference to Section 2.2). CT scans of patients with IPF, similar to CT scans of COVID-19 patients, show extensive patchy ground-glass regions with or without consolidations and the presence of pleural fluid. For this reason and considering that ENET was designed to be used with limited hardware resources (it was originally developed for image segmentation and recognition in self-driving car applications), and, as a result, benefits from rapid training and reduced training data requirements, we aim to investigate this approach for identifying COVID-19 infected subregions. Specifically, we customize this network by changing, in stage 3 as shown in Table 1, the ENET output of 128 × 64 × 64 to an output of 256 × 64 × 64. This implies that the C-ENET implementation is performing better than the original ENET, as shown in the DSC boxplots of Figure 3: although C-ENET and ENET methods are not statistically different, C-ENET shows an average DSC distribution greater and with less variability than ENET. Additionally, two solutions were implemented to overcome two typical issues in imaging data processing, namely unbalanced data (with reference to Section 2.3) and a limited number of labelled images (with reference to Section 2.4). Regarding the unbalanced data issue in CT images, COVID-19 infected regions are very small compared to the background characterized by a wide range of HU values. Consequently, DL networks tend to predict as target a CT region smaller than the true one. The Tversky loss function [27] was then used to assign a higher weight to the target voxels, unlike DSC which is the most used DL loss function. In this way, the network was able to learn more effectively how to recognize the target area, as reported in [28]. Regarding the issue of the limited number of labelled images, we address the problem through a five-fold cross-validation strategy. In this way, the whole CT dataset was randomly split into five folds: one-fold was used as the validation set and four folds were used as the training set. This process was repeated five times using each fold as the validation set and the others as the training set. As a result, five models were trained differently. The performance result was obtained by averaging the performance results of the validation datasets of the five models. Finally, two other state-of-the-art DL networks were evaluated in terms of segmentation accuracy, training and execution time, hardware and data requirements: UNET, the most widely used model for segmentation of biomedical image [24], and ERFNET [25] developed for the self-driving car task, such as ENET. All DLs were tested with the same public dataset of COVID-19 studies containing 199 CT images with labels provided by the Challenge [26] (with reference to Section 2.1) and a high-end HPC system equipped with GPU (NVIDIA QUADRO P4000 with 8 GB of RAM, 1792 CUDA Cores) (with reference to Section 2.4) which takes an average time of about 2–4 s to get the output for each study. Although the main limitation of this study was the limited number of cases, our results demonstrated the feasibility and effectiveness of C-ENET which showed a good segmentation accuracy with a DSC of approximately 75%. Furthermore, the choice of using a public dataset allows for easier reproducibility of the results and a clear reference for future studies in this research area: all images and labels are freely available for use by other researchers. Moreover, despite the relatively small number of cases, we obtained good results with an efficient solution both from the point of view of computational times and processing power requirements. Validation in a large image dataset will be performed to improve classification performance and to confirm our results, for example, using the datasets proposed in [32,33].

In conclusion, the clinical application of C-ENET can improve the way to assess the COVID-19 disease and open the way for a clinical decision-support system for risk stratification and patient management. In particular, the clinical application of C-ENET for automatic identification and segmentation of COVID-19 infected regions can provide tailored management for COVID-19 patients in discriminating the different degrees of disease aggressiveness. Finally, the application of this DL in a radiomics study will be described in a forthcoming paper.

## Figures and Tables

**Table 1:** Difference between ENET and C-ENET network architecture.

Name		Type	Stage	Output Size
initial			stage0	16 × 256 × 256
bottleneck1.0		Down-sampling	stage1	64 × 128 × 128
4 × bottleneck1.x			stage1	64 × 128 × 128
bottleneck2.0		Down-sampling	stage2	128 × 64 × 64
bottleneck2.1			stage2	128 × 64 × 64
bottleneck2.2		dilated 2	stage2	128 × 64 × 64
bottleneck2.3		asymmetric 5	stage2	128 × 64 × 64
bottleneck2.4		dilated 4	stage2	128 × 64 × 64
bottleneck2.5			stage2	128 × 64 × 64
bottleneck2.6		dilated 8	stage2	128 × 64 × 64
bottleneck2.7		asymmetric 5	stage2	128 × 64 × 64
bottleneck2.8		dilated 16	stage2	128 × 64 × 64
**ENET**	Repeat stage2, without bottleneck2.0		stage3	***128 × 64 × 64***
**C-ENET**	Repeat stage2, without bottleneck2.0		stage3	***256 × 64 × 64***
bottleneck4.0		Up-sampling	stage4	64 × 128 × 128
bottleneck4.1			stage4	64 × 128 × 128
bottleneck4.2			stage4	64 × 128 × 128
bottleneck5.0		Up-sampling	stage5	16 × 256 × 256
bottleneck5.1			stage5	16 × 256 × 256
fullconv			Final output	C × 512 × 512

**Table 2:** Performance of DL networks.

	DSC	VOE	VD	PPV	Sensitivity
C-ENET					
Mean	74.83%	39.01%	20.97%	76.26%	76.50%
±std	11.18%	13.67%	21.21%	10.01%	16.79%
±CI (95%)	3.51%	4.29%	6.66%	3.14%	5.27%
ENET					
Mean	72.28%	41.80%	27.60%	70.84%	77.10%
±std	13.44%	15.41%	38.35%	14.19%	15.95%
±CI (95%)	4.22%	4.84%	12.04%	4.45%	5.01%
ERFNET					
Mean	54.23%	60.56%	119.92%	48.65%	72.97%
±std	18.64%	17.66%	180.15%	20.67%	20.11%
±CI (95%)	5.85%	5.54%	56.54%	6.49%	6.31%

**Table 3:** ANOVA on the DSC showed statistical differences between DL methods.

ANOVA	F Value	F Critic Value	*p*-Value
C-ENET vs. ENET vs. ERFNET	22.010	3.076	*p* < 0.01

**Table 4:** Tukey HSD was used as a multiple comparison correction technique.

Tukey HSD	Q-Statistic	*p*-Value
C-ENET vs. ENET	10.659	0.7137408
C-ENET vs. ERFNET	75.402	0.0010053
ENET vs. ERFNET	86.062	0.0010053

**Table 5:** Comparison of computational complexity and performance of DL models.

Model Name	Number of Parameters		Size on Disk	Inference Times/Dataset		Training Times/Dataset
	Trainable	Non-Trainable		CPU (sec)	GPU (sec)	GPU (days)
C-ENET	793,917	11426	11 MB	16.857	4.026	4.22
ENET	363,069	8354	5.8 MB	12.833	3.505	3.47
ERFNET	2,056,440	0	25.3 MB	10.630	2.614	2.87

## Data Availability

CT Images in COVID-19—The Cancer Imaging Archive (TCIA) Public Access—Cancer Imaging Archive Wiki. Available online: https://wiki.cancerimagingarchive.net/display/Public/CT+Images+in+COVID-19#702271074dc5f53338634b35a3500cbed18472e0 (accessed on 25 March 2021).
